# Wavelength-Independent Excitation Bessel Beams for High-Resolution and Deep Focus Imaging

**DOI:** 10.3390/nano13030508

**Published:** 2023-01-27

**Authors:** Jing Wen, Zhouyu Xie, Shiliang Liu, Xu Chen, Tianchen Tang, Saima Kanwal, Dawei Zhang

**Affiliations:** 1 Engineering Research Center of Optical Instrument and Systems, Ministry of Education and Shanghai Key Lab of Modern Optical System, University of Shanghai for Science and Technology, No. 516 Jun Gong Road, Shanghai 200093, China; 2 Shanghai Institute of Intelligent Science and Technology, Tongji University, Shanghai 200093, China

**Keywords:** Bessel beams, metasurface, depth of focus, high resolution

## Abstract

Bessel beams are attaining keen interest in the current era considering their unique non-diffractive, self-healing nature and their diverse applications spanning over a broad spectral range of microwave to optical frequencies. However, conventional generators are not only bulky and complex but are also limited in terms of numerical aperture (NA) and efficiency. In this study, we experimentally develop a wavelength-independent Bessel beam generator through custom-designed metasurfaces to accomplish high resolution and large depth-of-focus imaging. These meta-axicons exhibit a high NA of up to 0.7 with an ability to generate Bessel beams with a full width at half maximum (FWHM) of 300 nm (~λ/2) and a depth of focus (DOF) of 153 μm (~261λ) in a broad spectral range of 500–700 nm. This excitation approach can provide a promising avenue for cutting-edge technology and applications related to Bessel beams for imaging along with a high axial resolution and an ultra-large depth of focus.

## 1. Introduction

Non-diffractive Bessel beams are a set of solutions to the free space Helmholtz equation discovered by Durin in 1987 [1]. They are termed Bessel beams due to their transverse intensity profile, which is defined by Bessel functions of the first kind. Bessel beams (BBs) have gained extensive research interest by exhibiting several exciting properties, for instance, self-healing and non-diffraction, and are of great significance in the broad spectrum of frequency ranges spanning from microwave to optical regimes for numerous applications, including tractor beams [2], particle trapping [3], and microscopy [4], as well as lithography and laser machining [5,6]. Likewise, Bessel beams are critical for high-power applications, for instance, wireless charging [7], microwave drilling [8,9], the formation of through-the-wall radar [10], and high-resolution radar imaging [11]. Accordingly, some straightforward and practical techniques for generating such beams are crucial.

Various methods have been employed to realize Bessel beams, such as computer-generated holograms [12], axicons [13], leaky waveguide modes [14], near-field plates [15], holographic screens [16], and transverse modes excited in circular waveguides [9], among others. However, Bessel beams generated by traditional methods are inappropriate for integration into optical devices due to their bulkiness, low efficiency, and limited depth of focus (DOF). In addition, conventional axicons have almost a constant NA in the visible regime caused by the weak dispersion of glass. Therefore, Bessel beams have a directly proportional FWHM that varies with the wavelength. Instead, subwavelength Bessel beams are typically generated by a composition of a high NA objective lens and an annular aperture [17], though in this configuration, a significant proportion of the light is choked by the aperture, making it less efficient. For generating higher-order Bessel beams, these approaches need additional phase-modulating elements, such as spiral phase plates or spatial light modulators.

The design approach in [9] demonstrates the excitation of transverse modes in a circular waveguide having a thickness greater than λ and an electrical size of 5.8λ × 5.8λ. The approach is not adaptable to integrated circuits and faces fabrication challenges. Comite et al. [18] demonstrated higher-order Bessel beams with twisted waves employing a radial line slot array (RLSA) antenna [19]. Bessel beams having an orbital angular momentum (OAM) were also considered with a fusion of (RLSA) and circular antenna arrays [11]. 

Despite the above-mentioned demonstrations, the excitation of high-efficiency circularly polarized (CP) non-diffracting BBs with a high NA and a large DOF is a key issue; meanwhile, they are critically important in sensing and manipulating biomolecules possessing chiral structures [20,21].

Metasurfaces, a developed group of ultra-thin planar optical components composed of subwavelength nanostructure arrays, have driven significant progress in recent years to tailor the wavefronts of electromagnetic (EM) waves. The realization of various exciting wave-manipulation properties is possible by suitably shaping the phase profiles of the metasurfaces [22], for instance, anomalous refraction/reflection [23,24,25], optical beams [26,27,28,29,30], beam array control [31,32,33], holograms [34,35,36], nonlinear devices [37], and metalenses [38,39,40,41,42,43]. Notably, transmissive metamaterial Huygens’ surfaces have been employed to generate Bessel beams in the microwave regime [44]. However, such an approach is limited to generating linearly polarized BBs with non-flat structures (due to the addition of a vertical split-ring resonator into the meta-atom design). Concurrently, Pancharatnam–Berry (PB) metasurfaces retain manifold benefits in controlling CP beams because of the frequency-independent nature of the generated phase distributions, straightforward design, and ease of fabrication as they just require the design of a single meta-atom [44,45,46].

Previously demonstrated high-efficiency Bessel beam generators in the optical frequencies [46] have used high NA meta-axicons to generate Bessel beams of different orders using TiO_2_ with high aspect ratio nanopillars, i.e., of 7:1, and the thickness of their design is wavelength comparable, i.e., 600 nm at λ_d_ = 405 and 532 nm. Nevertheless, besides the choice of material, a lower aspect ratio and subwavelength thickness have manifold advantages for the instant molding of optical wavefronts: easy fabrication and significantly reduced device size and weight against the bulky optical components. Moreover, the aspect ratio greatly affects the limitation of mass production. Realizing a metasurface with a high aspect ratio is more challenging. Therefore, the aspect ratio and efficiency should be simultaneously considered for efficient operations. However, as Si-based metasurfaces suffer from significant absorption loss in the visible range, they usually require a much higher aspect ratio to improve light manipulation capabilities. Therefore, it is crucial to achieve a low aspect ratio utilizing a material, such as silicon, and effectively reduce the fabrication challenges in the visible range. Hence, metasurface designers must continue to strike a balance between aspect ratio and efficiency.

In this study, considering this issue, we experimentally demonstrate excitation Bessel beams by employing custom-designed metasurfaces with subwavelength dimensions and ultra-large longitudinal depths. The approach has a low aspect ratio of 4:1 with a subwavelength thickness to reduce the fabrication challenges and provide miniaturization and integration to the optical devices. The NA of our excitation approach is 0.7, and not only can it generate BBs without requiring additional phase elements, but it also has a higher resolution and an ultra-large DOF of 153 μm (~261λ). Furthermore, utilizing the PB metasurface, the transverse field intensity profiles of BBs are made independent of the wavelength in the entire spectral range of 500–700 nm with a constant FWHM of 300 nm (~λ/2).

## 2. Materials and Methods

**Figure 1:**
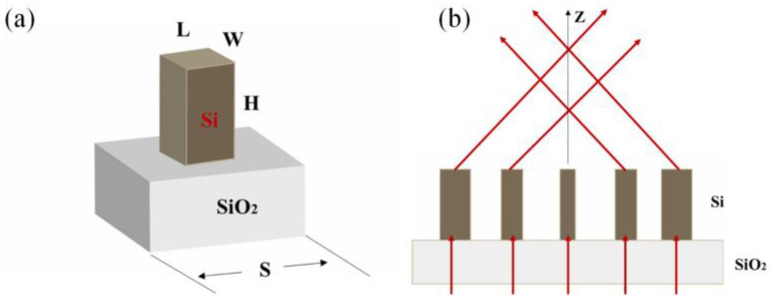
Schematic of Bessel beam generator. (**a**) Three-dimensional unit cell structure. (**b**) Excitation Bessel beams by an array.

The metasurface utilizes silicon rectangular nano-rods of the same size but rotating at different angles. *H* is the height, *L* is the length, and *W* is the width, which is arranged in a square lattice with period *S*. Pancharatnam–Berry phase is used to realize the required phase profile. Finite difference time domain (FDTD) applied by commercial software ‘FDTD Solutions’ (developed by Lumerical Solutions Co. Ltd., Vancouver, BC, Canada) [41] is used to carry out all the simulations. The optimized structural parameters of the nano-rod are *H* = 380 nm, *L* = 190 nm, *W* = 95 nm, and *S* = 285 nm at the design wavelength of λ_d_ = 587 nm. A simulated polarization conversion efficiency of 45.7% is achieved at λ_d_ = 587 nm. To evaluate the conversion efficiency, a silicon nano-rod array is arranged to deflect the light to a specific angle by generating the target phase profile and then calculating the conversion efficiency by dividing the total deflected light power by the input light power. In optimizing the parameters of the unit cell, perfectly matched layer (PML) boundary conditions are applied at the boundary perpendicular to the incident circularly polarized light, and periodic boundary conditions are used at other boundaries while simulating the metasurface arrays; PML boundary conditions are used. Figure 1 shows the schematic diagram of the excitation Bessel beams.

In order to generate a zero-order Bessel beam, each structural unit of the metasurface needs to polarize the beam at the same angle, and the position of each structural unit needs to have a specific radial phase gradient. The required radial phase profile is φ(r), having a phase gradient:

(1) $$\frac{d\varphi }{dr}=\frac{2\pi }{\lambda_{d}}\sin(\theta )$$

As stated by the generalized Snell’s law [24], all light rays at the design wavelength *λ*_d_ should refract by the same angle, i.e., *θ*, wherein sin(*θ*) is the NA. Merging it in Equation (1) gives:

(2) $$\varphi (x,y)=2\pi -\frac{2\pi }{\lambda_{d}}\cdot \sqrt{x^{2}+y^{2}}\cdot \mathrm{NA}$$

where $\sqrt{x^{2}+y^{2}}=r$. The phase profile is given by the rotation angle of each nano-rod at the position (x,y). For left circularly polarized incidence light, the rotation angle is θ(x,y)=φ(x,y)/2.

**Figure 2:**
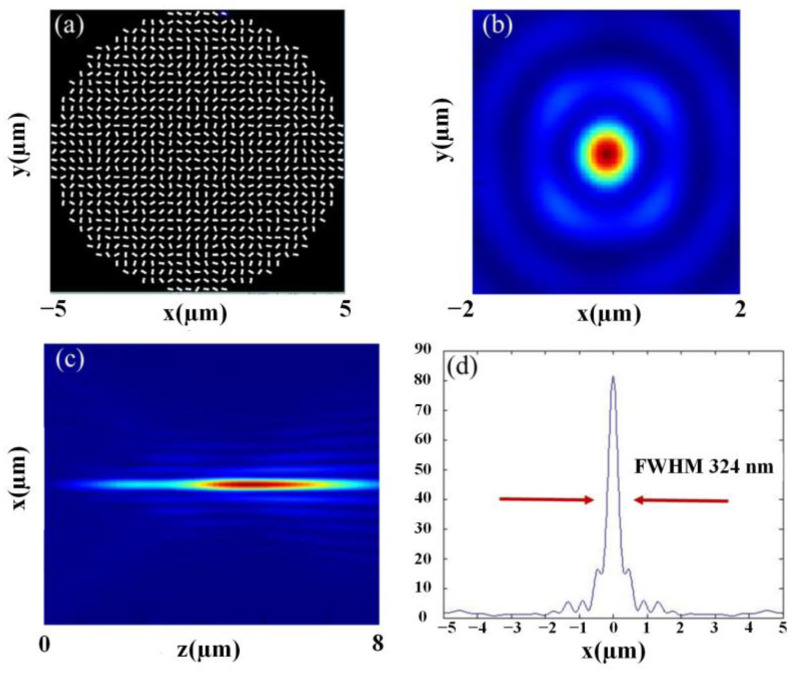
Simulation results of 10 μm diameter metasurface, NA = 0.7; (**a**) schematic diagram of metasurface; (**b**) optical field distribution of Bessel beam cross-section; (**c**) x-z plane light field distribution; and (**d**) *x*-axis intensity distribution curve.

After obtaining the phase profile of the BBs, the metasurface is modeled and simulated. The simulation results are exhibited in Figure 2.

Shown in Figure 2a is the schematic of the metasurface. Figure 2b is the light field distribution of the Bessel beam, Figure 2c is the light field distribution of the axial cross-section, and Figure 2d is the intensity profile of the BB in the *x*-direction with its FWHM of 324 nm.

## 3. Fabrication and Experimental Results

### 3.1. Fabrication

The metasurface is achieved by electron beam lithography and lift-off methodology. First, a uniform silicon film with a thickness of 380 nm is deposited on a silicon dioxide substrate by a sputtering machine. Then, a 350 nm thick resist layer (ZEP520) is spin-coated on the wafer, and the hot plate is baked at 180 °C for 1 min. The electron beam lithography system (JBX6300fs by JEOL, Peabody, MA, USA) is used to operate the E-beam lithography at an acceleration voltage of 100 keV. Afterwards, the sample is developed at room temperature in an amyl acetate solution for 65 s. For etching, an inductively coupled plasma reactive ion etching machine (ICP-RIE by CORIAL, a Plasma-Therm Company, Bernin, France) is used, and a relatively large-sized nano-rod array of 300 μm is realized through E-beam lithography. Considering the fabrication constraints, the pitch size is adjusted to 285 nm.

### 3.2. Optical Characterization

**Figure 3:**
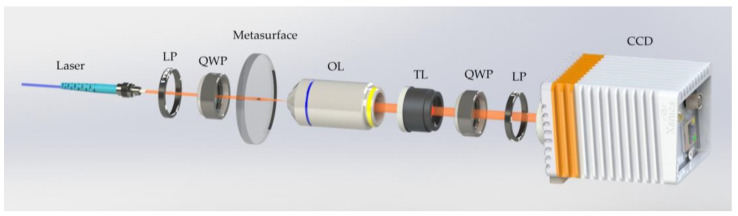
Schematic of the optical setup for the experimental validation. LP (linear polarizer); QWP (quarter-waveplate); OL (objective lens); TL (tube lens); CCD (charge-coupled device camera).

**Figure 4:**
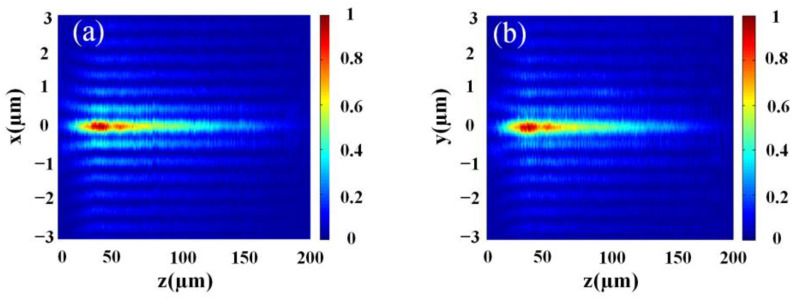
Light field profiles of the BBs with NA 0.7 at the wavelength λ_d_ = 587 nm along the longitudinal direction; (**a**) x-z plane (**b**) y-z plane.

The optical setup for the experimental validation of light field distribution of the excitation BB is shown in Figure 3. A collimated beam from an NKT supercontinuum light source (SuperK EXTREME EXR-15, by NKT Photonics, Boston, MA, USA) is deployed to the left circularly polarized beam through an LP (LPVIS100 by Thorlabs, Newton, NJ, USA) and a QWP (AQWP05M-600 by Thorlabs, Trenton, NJ, USA) (passing linearly polarized light through a quarter-waveplate with its axes at 45° to its polarization axis will convert it to circular polarization), and the metasurface is vertically illuminated by the incident light. To begin with, the transmitted light field of the generated BBs is magnified by a 100× Leica objective of NA = 0.85 and a TL of *f* = 300 mm; eventually, the imaging of these fields is performed using a CCD camera (DH-HV3151UC by China Daheng (Group) Co., Ltd. Beijing, China). Another QWP (circularly polarized light can be converted into linearly polarized light by passing it through a quarter-waveplate) and LP are placed between the TL and the CCD camera to filter out RCP light, that is, inverse CP transmitted light. The OL and the TL are fixed on the XY-scanning stage (MLS230-1 by Thorlabs, Trenton, NJ, USA), moving at a step-size of 1 μm along the longitudinal direction, thereby recording the magnified transverse fields at the discrete positions. By using the customized LABVIEW program, the movement of the motorized stage and the time of the CCD-captured image are synced. Finally, the 2D x-y field distribution slices, recorded per discrete longitudinal point along the propagation direction of the incident beam, are superimposed and then rebuilt into 3D field patterns, wherefrom the longitudinal x-z and y-z field profiles (as shown in Figure 4a,b) and the measured light field distribution of the cross-section of the BBs are extracted. 

**Figure 5:**
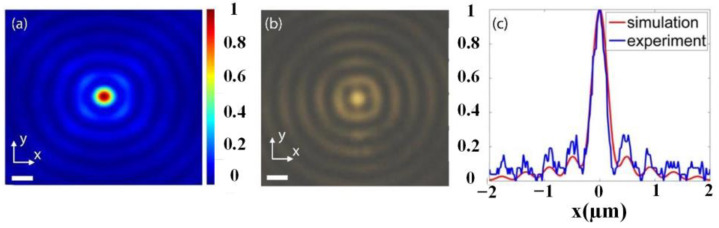
Light field intensity profiles on the transverse cross-section of the BB at λ_d_ = 587 nm; (**a**) numerically simulated; (**b**) experimentally recorded; (**c**) intensity profiles of (**a**) and (**b**) along the *x*-axis.

The measured DOF of the BB is as large as 153 μm (~261λ) by fabricating an ultra-large array of 300 μm. The numerical simulation and experimental validation of light field profiles on the transverse cross-section of the BB at λ_d_ = 587 nm are shown in Figure 5, where Figure 5a is the numerically simulated and Figure 5b is the experimentally recorded cross-section light field distribution of the BB, while the horizontal profiles of Figure 5a,b are compared in Figure 5c. Apparently, the simulated light field distribution of the Bessel beam is validated experimentally. 

**Figure 6:**
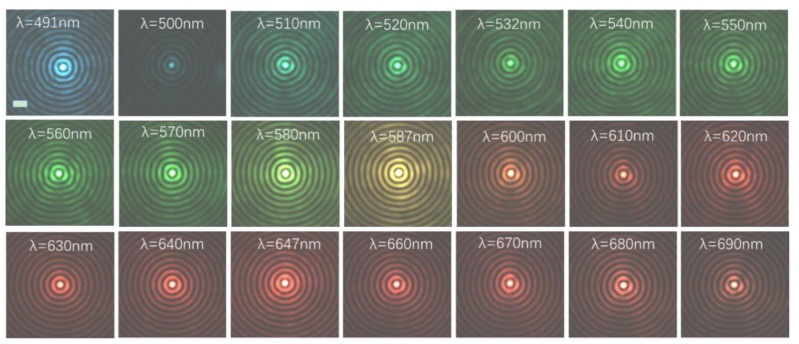
Experimentally measured intensity profiles of Bessel beams generator with wavelength-independent characteristics. NA = 0.7 and design wavelength λ = 587 nm. (Measured intensity profiles at wavelengths λ = 491, 500, 510, 520, 532, 540, 550, 560, 570, 580, 587, 600, 610, 620, 630, 640, 647, 660, 670, 680, and 690 nm). Scale bar: 1 μm.

The experimentally measured light field distribution of the cross-section of the BBs at various wavelengths over a broad spectrum of 500–700 nm is shown in Figure 6.

**Figure 7:**
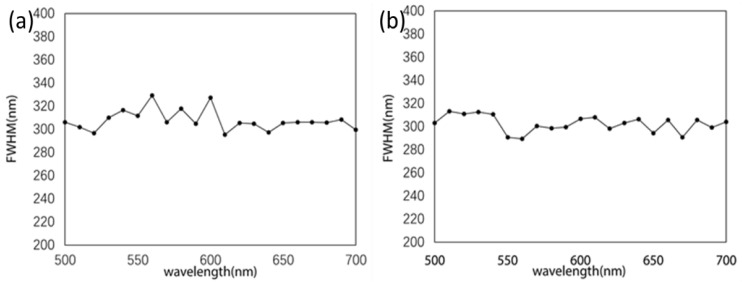
Experimental measurement of FWHM of subwavelength Bessel beam. (**a**) FWHM in the *x*-direction and (**b**) FWHM in the *y*-direction.

This certainly demonstrates the weakly varying intensity profile for diverse wavelengths, confirming the wavelength-independent characteristic of the Bessel beam and validating that the quality of the generated Bessel beam is consistent with the design wavelength. To further analyze the subwavelength characteristics of the generated Bessel beam, the full width at half maximum (FWHM) is measured, which is 300 nm. The experimental records are shown in Figure 7.

Figure 7 displays the FWHM of the BBs measured in a broad spectral range of 500–700 nm in the (a) *x* and (b) *y* directions. The experimental results show that the FWHM of the Bessel beams is near 300 nm in both directions, which is comparable to the theoretical value of 324 nm.

It is worth noting that, traditionally, the FWHM of Bessel beams is proportional to the wavelength and increases accordingly. The transverse intensity profile is obtained by the factor $k_{r}=\frac{2\pi }{\lambda }\cdot \mathrm{NA}$. In our case, $\mathrm{NA}=\frac{\lambda }{2\pi }\nabla \varphi (x,y,\lambda )$, where *φ* follows Equation (2). Hence, *k_r_* just depends on the phase gradient ∇φ(x,y,λ) that can be made wavelength-independent by utilizing the PB phase, and the FWHM of the Bessel beam can be kept unchanged in the entire operating wavelength range, showing a wavelength-independent transverse intensity distribution characteristic. Thereon, the phase gradient is constant, and the NA is just related to the wavelength λ [46]. Our generated BBs have a lower aspect ratio with a high resolution and subwavelength thickness. Furthermore, due to the highly concentrated energy of the Bessel beam, it has a smaller spot size than the Gaussian focal spot with the same numerical aperture and can easily achieve diffraction-limit focusing. There are various limitations to traditional Bessel beam generators, such as low energy utilization, complex structure, limited numerical aperture, and wavelength-dependent spot size. The excitation approach demonstrated in this study can address these limitations and will play an important role in biological super-resolution microscopic imaging.

## 4. Discussion

Previously demonstrated high-efficiency Bessel beam generators have the limitations of wavelength comparable thickness by using high-aspect-ratio nanopillars. However, lower aspect ratio and subwavelength thickness have various advantages, including ease of fabrication and miniaturized devices compared to bulky optical components. In addition, the aspect ratio greatly affects the limitation of mass production. Therefore, it is crucial to achieve a low aspect ratio and high efficiency simultaneously and reduce the fabrication challenges for efficient operations in the visible range. Hence, in this study, we effectively developed wavelength-independent high-resolution excitation Bessel beams with a large depth of focus and a low aspect ratio of 4:1 with subwavelength thickness, based on PB phase metasurfaces. Given this, we carried out numerical simulations and then processed the samples by using the electron beam etching method. Afterwards, an experimental optical path was developed to verify the processed samples to further analyze and validate the results. Along with a high NA of 0.7, the FWHM of Bessel beams was displayed to be as small as ~300 nm, i.e., (~λ/2), in the broad spectral range of 500–700 nm. The FWHM was retained for a remarkably large distance, i.e., a depth of focus of 261λ. The experimental and simulation results are all consistent and developed a Bessel beam generator that has no relationship between beam size and wavelength. This excitation approach can be employed to simplify and minimize the design of light-sheet microscopies and eventually be utilized to dynamically record imaging with the super spatial resolution and an ultra-large DOF.

## Data Availability

The data presented in this study are available in the article.
